# Acceptance and Potential Impact of the eWALL Platform for Health Monitoring and Promotion in Persons with a Chronic Disease or Age-Related Impairment

**DOI:** 10.3390/ijerph17217893

**Published:** 2020-10-28

**Authors:** Francesco Infarinato, Stephanie Jansen-Kosterink, Paola Romano, Lex van Velsen, Harm op den Akker, Federica Rizza, Marco Ottaviani, Sofoklis Kyriazakos, Beatrix Wais-Zechmann, Markus Garschall, Stefano Bonassi, Hermie J. Hermens

**Affiliations:** 1 IRCCS San Raffaele Pisana, 00166 Rome, Italy; francesco.infarinato@sanraffaele.it (F.I.); laboratoriocognitivoroma@gmail.com (F.R.); marco.ottaviani@sanraffaele.it (M.O.); stefano.bonassi@sanraffaele.it (S.B.); 2 Roessingh Research and Development, 7522 AH Enschede, The Netherlands; s.jansen@rrd.nl (S.J.-K.); L.vanVelsen@rrd.nl (L.v.V.); H.opdenAkker@rrd.nl (H.o.d.A.); H.Hermens@rrd.nl (H.J.H.); 3 Department of Business Development and Technology, BTECH, Aarhus University, 7400 Herning, Denmark; sofoklis@btech.au.dk; 4 Innovation Sprint, 1200 Brussels, Belgium; 5 AIT Austrian Institute of Technology GmbH, 1210 Wien, Austria; Beatrix.Wais-Zechmann@ait.ac.at (B.W.-Z.); Markus.Garschall@ait.ac.at (M.G.); 6 Department of Human Sciences and Quality of Life Promotion, San Raffaele University, 00163 Rome, Italy

**Keywords:** eHealth, telemonitoring, telerehabilitation, ICT platform, acceptance of technology, user experience, MCI, COPD, chronicity, frailty, patient empowerment, pervasive healthcare, health apps

## Abstract

Pervasive health technologies can increase the effectiveness of personal health monitoring and training, but more user studies are necessary to understand the interest for these technologies, and how they should be designed and implemented. In the present study, we evaluated eWALL, a user-centered pervasive health technology consisting of a platform that monitors users’ physical and cognitive behavior, providing feedback and motivation via an easy-to-use, touch-based user interface. The eWALL was placed for one month in the home of 48 subjects with a chronic condition (chronic obstructive pulmonary disease—COPD or mild cognitive impairment—MCI) or with an age-related impairment. User acceptance, platform use, and potential clinical effects were evaluated using surveys, data logs, and clinical scales. Although some features of the platform need to be improved before reaching technical maturity and making a difference in patients’ lives, the real-life evaluation of eWALL has shown how some features may influence patients’ intention to use this promising technology. Furthermore, this study made it clear how the free use of different health apps is modulated by the real needs of the patient and by their usefulness in the context of the patient’s clinical status.

## 1. Introduction

Pervasive health technology—ensuring a continuous flow of information from the comfort of the user’s home—allows persons to claim a more active role in the management of their health [1]. Radio-frequency identification transponders (RFID tags), domotics devices, wearable sensors, and smartphones are creating connections between the physical environment of patients and caregivers using the internet and intelligent computing. As such, pervasive technology, represents a perfect framework for eHealth, i.e., the organization and delivery of health services and information using the internet and related technologies, as defined by Eysenbach [2]. Several examples of services that rely on pervasive technology are available, to monitor physiological signals with a network of sensors [3], to allow co-presence in the school class [4], or to track end-user’s caloric intake and expenditure via on-body sensors and mobile applications [5].

End-user populations that require constant health monitoring and/or are prone to use new technologies willing to improve their health are those that may especially benefit from pervasive technology. These populations offer the best conditions for viable business models and durable services. People with age-related impairments (ARI), chronic obstructive pulmonary disease (COPD), or mild cognitive impairment (MCI) are suitable examples of population groups fitting this description.

Older persons with ARI start losing functional capacity in the physical, cognitive, or psychological domain, and have an increased risk of frailty [6]. COPD is a chronic lung disease which is characterized by an enhanced inflammatory response in the airways and lungs. This disease has a progressive course, especially in persons who continue to smoke [7], and dramatically influences quality of life due to shortness of breath (dyspnoea), chronic cough and chronic sputum production [8]. Subjects affected by COPD often become inactive due to dyspnea during physical activity [9]. MCI, beyond age-related symptoms, is the first step of transition from healthy ageing to pathological dementia. In many cases persons with MCI preserve cognitive and functional abilities and this allows them to carry out normal daily activities [10]. The Diagnostic and Statistical Manual of Mental Disorders [11] refers to this stage as mild neurocognitive disorder. Individuals belonging to these groups suffer from chronic conditions which are very common in the ageing population and require constant monitoring and effective strategies aimed at contrasting physical and cognitive decline.

Despite the great potential that pervasive health technology offers for persons with progressive physical or cognitive disorders (such as ARI, COPD, or MCI), there is still a lack of success stories. A limiting factor is surely the alleged limited interest of end-users in pervasive health technology services. A recent study which conducted focus group interviews with people having chronic conditions [12] indicated that, contrary to the common belief, older adults are willing and capable of engaging with mobile health technology, and the same holds for the pervasive technology which was found to be accepted equally well among younger and older end-users [13]. In both cases the main limiting factor is the cost of new technologies. Persons with COPD were extremely positive about the access to eHealth programs, although the limited subjective benefits limit the confidence in these technological tools [14]. The subjective benefits could be enhanced by tailoring the technology to the needs of persons with COPD, as suggested by [14,15]. Persons with MCI and especially their caregivers are prone to accept the use of such new telecare technologies, although some technical and ethical concerns remain [16].

The application of pervasive health technology in real life experienced some major pitfalls. After reviewing the literature, Orwat and colleagues [17] concluded that most experiences with pervasive health technology were in a prototypical stage, that logistic details, privacy concerns, and financial issues were rarely considered, and eventually, that more clinical, economic, and user studies were required. More recently, Loncar-Turukalo and colleagues [18] identified six gaps or key concepts in the literature concerning the design and evaluation and user preferences of pervasive health technology. The lack of objective and reliable data collected during the studies, the limited implementation of longitudinal studies, and the lack of studies that focus on ‘more challenging’ end-user groups, such as children and older adults, were the most critical issues.

In this article, we report on the real-life experience with eWALL—a home care platform that monitors the end-user’s physical and cognitive behavior, and provides feedback and motivation via an easy-to-use, touch-based user interface [19] presented on a large screen. Information is collected, stored and analyzed in the cloud, enabling the platform to keep healthcare professionals or family members in the loop if desired. The aims of this study were to understand the end-user acceptance and determine the potential health benefits of eWALL for these patients. The study has a longitudinal approach based on multiple data sources (quantitative surveys, qualitative interviews, data logs). The main research question was how a pervasive health technology, such as eWALL, is accepted and used by persons with a chronic disease or age-related impairments and how it impacts their physical and mental health. The study was designed also to evaluate (1) factors influencing the intention to use eWALL by patients with a chronic disease or age-related impairments, (2) the approach of patients with a chronic disease or age-related impairments to use eWALL when implemented in their home environment, and (3) the potential clinical effect of using eWALL by patients with a chronic disease or age-related impairments.

Answering these questions will allow us to fill the knowledge gaps discussed above with a result in practical design advice for future studies with pervasive health technology for patients with chronic diseases. The framework of the paper will be structured as follow: (i) after a detailed description of the eWALL platform technology and its installation at patients home, (ii) the measurements protocol adopted to evaluate the impact of tele-monitoring in the life of frail elderly will be exposed; (iii) results will be study in deep and accurately show supported by statistical algorithms aimed at (iv) verify their reliability, discuss what parameters influenced acceptance and utilization of such kind of services in the field of healthcare, pointing also on limitations and other issues; (v) finally conclusive statements will be drawn.

## 2. Materials and Methods

**Figure 1:**
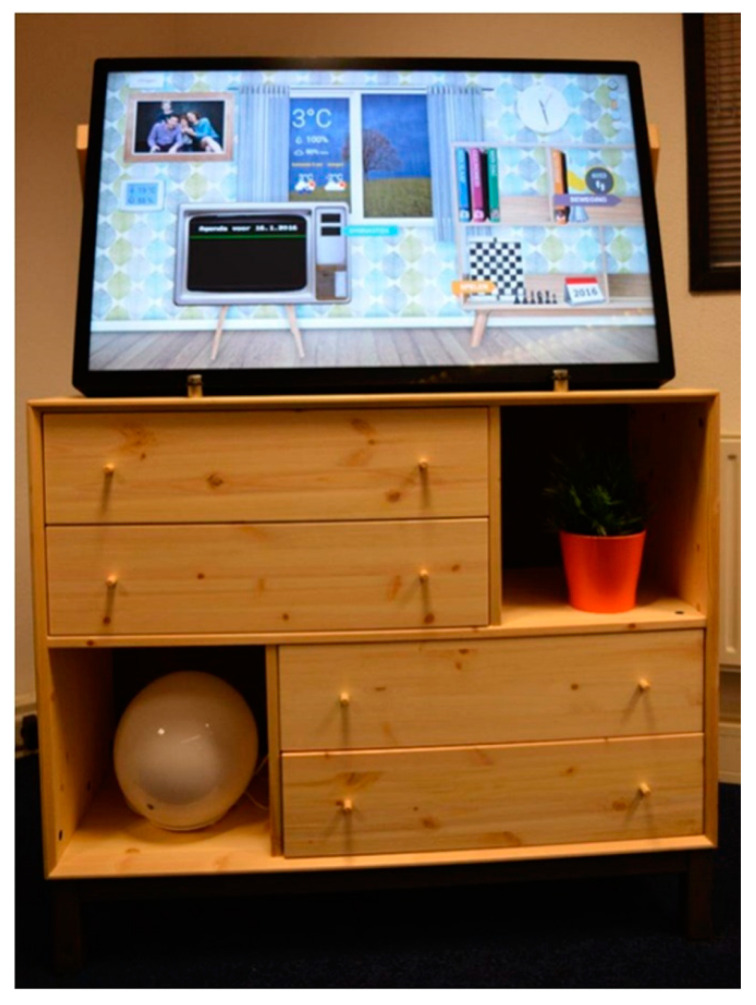
The eWALL home device, with a 40′′ touch screen presenting the user interface, mounted on a cabinet that hides essential hardware.

The eWALL platform [19] is a technological service that consists of a centralized, cloud-based server architecture (where data is collected in one central repository and processed subsequently), and front-end user interfaces for both primary users (people with a chronic disease or age-related impairments) and secondary users (healthcare professionals). A 40” touch-screen, installed into the living room of the primary users, serves as an interactive interface to the eWALL platform (see Figure 1).

The user interface is mounted on a cabinet which hides away hardware responsible for running the interactive home software, and collecting and forwarding sensor data to the cloud. Behavioral (steps, stairs, sleep, calories burnt), environmental (temperature and humidity optionally monitored in the various rooms of the house), and biometric data (blood pressure monitor, oxygen saturation monitoring) are collected wirelessly (Bluetooth protocols)from activity tracker, medical monitoring devices and domotic sensors.

**Figure 2:**
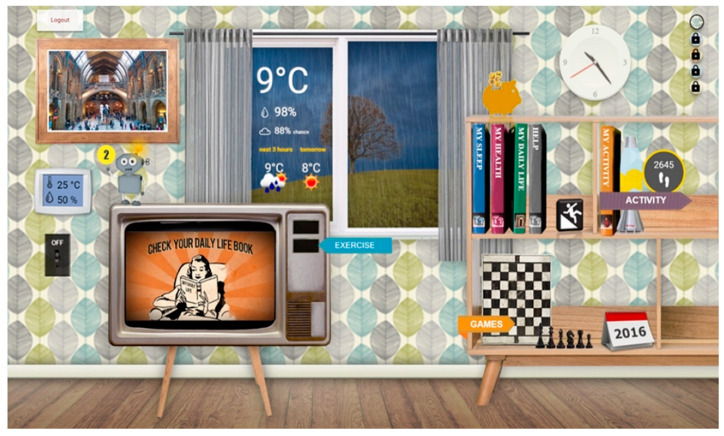
The eWALL’s main user interface, boasting a skeuomorphic design—mimicking a real wall with interactive elements.

The touch-based user interface provides access to various health and wellbeing applications. The interface design visualizes a virtual living room (Figure 2), with each object showing information or acts as a shortcut to the different applications.

The photo frame shows pictures that family members or friends can upload through an online service. The window indicates the current and upcoming weather at the user’s location. The lava lamp shows the number of steps taken on a day and changes its color from red to yellow and green to indicate to users how close they are to reaching their daily step goal.

The set of “book applications” (My Sleep, My Health, My Daily Life, and My Activity) provides information about the user’s lifestyle, showing daily records of sleeping or activity behavior, or sensor-based information like blood pressure. A game board set in the bookcase gives access to a set of games (e.g., memory training, or puzzles) for cognitive rehabilitation, while a specially designed calendar can help users in structuring their daily life by facilitating the scheduling of everyday activities like having breakfast or taking a shower using drag-and-drop functions. The television shows “advertisements” for healthy behavior or can be used to access a personalized physical rehabilitation program with increasing difficulty level, giving the possibility to rate difficulties and pleasure for each exercise. A virtual embodied agent—Robin the Robot (on top of the television), assists the user in using the applications, and gives tailored advice on achieving daily physical activity goals.

### 2.1. Participant Recruitment

Participants were recruited in Austria, Denmark, Italy, and the Netherlands between April and October 2016.

- People with age related impairments were recruited in Denmark and Austria and were included if they were over 65 years of age and had a score below 65 on the physical functioning scale of the Short Form 36 (SF36-pfs). Exclusion criteria for these participants were physical and/or cognitive impairments that do not allow proper use of eWALL, lack of independence in functional abilities and an inability to read and speak the language in which eWALL is offered (Italian, Dutch, German, English, Danish);
- Patients with COPD were included in Denmark and in The Netherlands if they were clinically diagnosed with COPD (GOLD stage 2, 3, or 4) and when their disease was stable (i.e., no infection or exacerbation in the four weeks prior to inclusion). Exclusion criteria for these participants were: having other diseases that influence bronchial symptoms or the need for oxygen therapy, impaired hand function or disorders causing an inability to use eWALL, and the inability to read and speak the language of the clinical site;
- Subjects with MCI were included in Italy if they were over 65 years of age, had a Mini Mental State Examination (MMSE) score adjusted for age and education above 23, had a Clinical Dementia Rating (CDR) score of 0.5, had a pathological score of 3 in one episodic memory test or in one of other function and had normal scores with all the other battery tests. These participants were excluded if they were over 80 years of age, had presence of a physical impairment that does not allow the proper use of the eWALL, experience lack of independence in functional abilities and were unable to read and speak the language of the clinical site.

This study was conducted according to the principles of the Declaration of Helsinki (64th WMA General Assembly, Fortaleza, Brazil, October 2013) and in accordance with The Directive 95/46/EC of the European Parliament and of the Council of 24 October 1995 (amended with the regulation (EC) No 1882/2003) on the protection of individuals with regard to the processing of personal data and on the free movement of such data. Local Ethics Committees approved or received communications with the study protocol (METC TWENTE-P15-24; CE IRCCS San Raffaele Pisana 5/2016). All patients provided written informed consent.

### 2.2. Procedure

Before inclusion, interested participants received a clear explanation of the purpose of the study and all the technical aspects concerning eWALL and its installation. During the first week of the study, technicians installed the eWALL in the participant’s home and explained all the functionalities of the eWALL. Participants and informal caregivers received a training on how to navigate the main screen and various applications. Pre-test assessments were conducted at the participant’s home after installation. These assessments were performed by professionals with a clinical background. Participants had access to eWALL for a period of four weeks. Participants and their informal caregiver had the possibility to keep in touch with technical staff; moreover, a devoted telephone line was available, also out of office hours. Apart from this, the eWALL contained a Help application with information about the various applications and an overview of frequently asked questions. At the end of the four weeks home intervention period, the eWALL was uninstalled and participants completed the post-test assessment.

### 2.3. Measurements

#### 2.3.1. Technology Acceptance

During the early design and implementation phases of the platform, researchers applied different methods for understanding end-user acceptance of an eHealth technology [20]. In the experimental phase, end-user acceptance of eWALL was assessed by means of a questionnaire with 7 levels summated rating scales, based upon the Technology Acceptance Model (TAM) [21]. TAM originates from the 1980s and has been used numerous times to assess and explain the acceptance of new technology. We expanded TAM with factors that have been found to shape the user experience of eHealth technology: Enjoyment [22], Aesthetics [23], Control [24], and Trust in technology [25]. We hypothesize that these factors affect the core factors of TAM that explain the intention to use (perceived usefulness and ease of use). The scale for enjoyment was based on Van der Heijden [26], aesthetics was taken from Lavie and Tractinsky [27], control and the intention to use were derived from Van Velsen et al. [28], Trust in technology was based on McKnight et al. [29], ease of use was taken from Venkatesh et al. [30]. We developed a scale for perceived usefulness ourselves, as we wanted this scale to specifically target the goals of using eWALL. It is worth to underline that the score associated with a domain is obtained by the sum of all the domain-related items divided by the number of items (simple average). As we used a 7-levels Likert scale, a score of 4 indicated that the person was indifferent to the question or that the person had no clear opinion about that issue. A higher score means a positive attitude towards the categories and a lower score, a lower attitude.

#### 2.3.2. Use of Technology

To assess the use of the device and its individual applications, an interaction logging service was developed that keeps track of user’s interactions with the user interface. The analysis of data logs was focused on the following aspects:

(1) Interaction with eWALL. We grouped all interactions that occur on a specific day for a specific user and analyzed the data over time.
(2) Interaction with the eWALL applications. We calculated for the individual participants as well as for all participants together, how many interactions they had with each application.
(3) Frequency of use of each of eWALL applications. We counted for every participant and for every day whether or not each application was used. For example, if a participant used the My Activity Application on 21 different days, while he had access to eWALL for 28 days, we define his application use for the My Activity Application as 21/28 = 75%.
(4) Time of day of using eWALL. We calculated for each half-hour of the day, how many interactions with eWALL took place in total.

In order to triangulate the quantitative data in the data logs [31], we conducted semi-structured interviews with each participant at the end of each trial period. A predefined interview scheme with 20 questions about technical problems that occurred while working with eWALL, the use and appreciation of the different functionalities (e.g., activity tracking, the sleep diary, cognitive games, environmental information), and the appreciation of eWALL as a whole.

#### 2.3.3. Potential Clinical Effect

The potential clinical effect was defined by the Short Form (36) Health Survey (SF-36) [32]. The SF-36 was used to assess the perceived quality of life. This questionnaire consists of 36 items, divided into two domains: the physical domain (physical function, role functioning, bodily pain and general health) and the mental domain (mental health, emotional role functioning, social function and vitality). This questionnaire was completed by the participants before (pre-test) and after (post-test) the use of eWALL.

#### 2.3.4. Analyses

Statistical analyses were performed using SPSS, version 19.0 (IBM, Armonk, NY, USA). All outcome measures were inspected for a normal distribution of data, using histogram plots with normality curves and normal probability plots. These determined our selection of appropriate statistical tests. Mean scores ± standard deviation (SD), or median with range were calculated for each of the outcome measures. For statistical analysis, the level for significance was set at α < 0.05.

To determine the reliability of the psychometric measurement scales, Cronbach’s alpha was calculated for each factor, whereby we considered a score of ≥0.70 to be good reliability [33]. As prerequisite for conducting regression analyses among the technology acceptance factors, we determined the correlation (Pearson’s) among all these factors. Based on the correlation analyses, backward stepwise regression analyses (with a probability of F-to-remove ≥0.10) were conducted because we do not have any a priori assumptions about the causalities among the factors. When the data concerning the potential clinical effects was normally distributed, effects over time were assessed via univariate testing. Mean values were compared with paired student’s *t* tests. For the analysis of the interviews, we follow the method as defined in Patton [34] and applied Question Analysis (an approach focused on participants’ responses to questions, related to specific screens or functionalities of eWALL). This means that each interview was written out in full, two researchers listed the different responses to each question, and then determined how often each response was given. In the case of disagreement, they discussed the issue until agreement was reached.

## 3. Results

### 3.1. Participants

In total, 50 participants were enrolled in the study: 17 participants with MCI (all from Italy), 24 participants with COPD (21 from The Netherlands and 3 from Denmark) and 10 participants with age-related impairments (5 from Austria and 5 from Denmark). Two MCI participants and one COPD participant decided to leave the study for personal reasons. After installation, two participants with COPD dropped-out due to an increase in stress levels, attributable to their involvement with the study. One participant with ARI decided to leave the study due to technical difficulties related to the immaturity of the prototype. Main characteristics of the participants are described in Table 1.

### 3.2. Acceptance

#### 3.2.1. Reliability Analysis

Not all the participants completed the whole intervention time. The evaluation of the acceptance was based on data from 15 MCI, 16 COPD, and 5 ARI participants. To determine the reliability of the Technology Acceptance measurement scales Cronbach’s alpha for the different rating scales was calculated. Based on a minimum threshold of 0.7, it can be stated that all scores were from good to excellent. In Table 2 the number of items and Cronbach’s alpha values are reported for each domain. Nevertheless it could be expected that more important domains are sustained by a major number of questions, the numerosity of items is not directly linked to the significance of the domain. For example, the domain related to Aesthetics included more fields of interest, such as creativity, clarity, balance and transitions/animations/effects.

Table 3 displays the mean scores for the different technology acceptance factors per participant group and overall. All factors were appreciated positively or moderately by the ARI and MCI groups, while COPD patients generally did not appreciate eWALL in any factors.

#### 3.2.2. Correlation Analysis

The calculation of the linear correlation was a prerequisite for conducting regression analyses, Pearson’s correlation coefficient was computed among all technology acceptance factors. Results described in Table 4 show that all factors were significantly positively related (*r*’s from 0.53 to 0.86).

#### 3.2.3. Regression Analysis

Three backward linear regression analyses were conducted on the following dependent variables, namely: ease of use, perceived usefulness, and intention to use.According to results from the correlation analyses, Enjoyment, Aesthetics, Controllability, and Trust in technology were included in the model explaining the ‘ease of use’. The first model resulted in an R² of 0.81, and included Aesthetics (β = 0.38, t = 3.48. *p* < 0.01) and Controllability (β = 0.59, t = 5.37. *p* < 0.001). For the dependent variable ‘Perceived usefulness’, we included the factors Enjoyment, Aesthetics, Controllability, Trust in technology, and Ease of use. The model resulted in an R² of 0.50, and included four not significant predictors: Enjoyment (t = 0.75, *p* = 0.46), Ease of use (t = 0.37, *p* = 0.71), Controllability (t = 0.18, *p* = 0.86), and Aesthetics (t = −0.21, *p* = 0.82). Trust in technology affected Perceived usefulness significantly (β = 0.52, t = 2.56, *p* < 0.05). The model to explain ‘Intention to use’, included the Perceived usefulness and the Ease of use. The first run resulted in an R² of 0.58. Both predictors significantly affected the Intention to use (Ease of use: β = 0.28, t = 2.08, *p* < 0.05; Perceived usefulness: β = 0.57, t = 4.28, *p* < 0.001).

**Figure 3:**
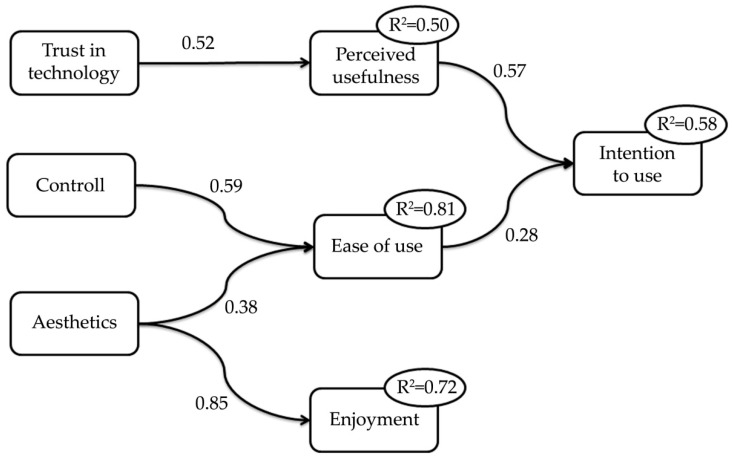
Diagram summarizing the regression analyses on Technology Acceptance for eWALL.

A fourth backward linear regression analysis was conducted to study the influence of Aesthetics on Enjoyment, showing a significant association, i.e., β = 0.85, t = 9.28, *p* < 0.001. Figure 3 shows the diagram summarizing the four backward linear regression analyses.

### 3.3. Use

**Figure 4:**
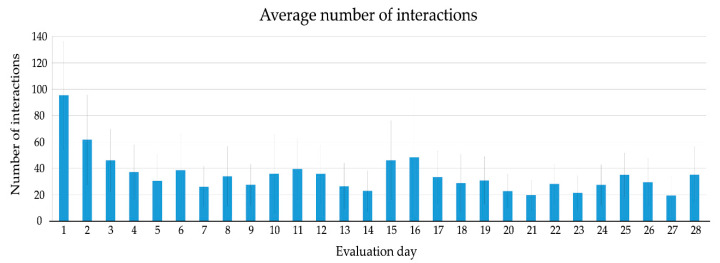
Average number of interactions on evaluation day (values averaged over all active users on that day).

After installation, 48 participants used the eWALL on the first day and kept using the system for the first 6 days. The first dropouts occurred on the 7th and 8th day. After two weeks, 85% of the participants were still using eWALL and after three weeks, 67% of the participants were still active. The average number of interactions of the participants with eWALL are plotted in Figure 4. Participants had, on average, 95 interactions with the device (S.D. = 82), the trend shows a clear drop after the first 5 days of use, after that a quite constant level appears between 20 and 40 interactions a day, indicating that participants showed a regular and stable usage pattern over the course of the evaluation period (4 weeks). The average number of interactions in the first week (47.8) was slightly higher than in the consecutive weeks. The remaining weeks showed a stable pattern of use (on average, 31.6 daily interactions in the second week, 32.7 in the third, and 28.0 the fourth).

As each interaction with eWALL was annotated, a list of the applications most frequently used by the participants was generated (see Table 5).

**Figure 5:**
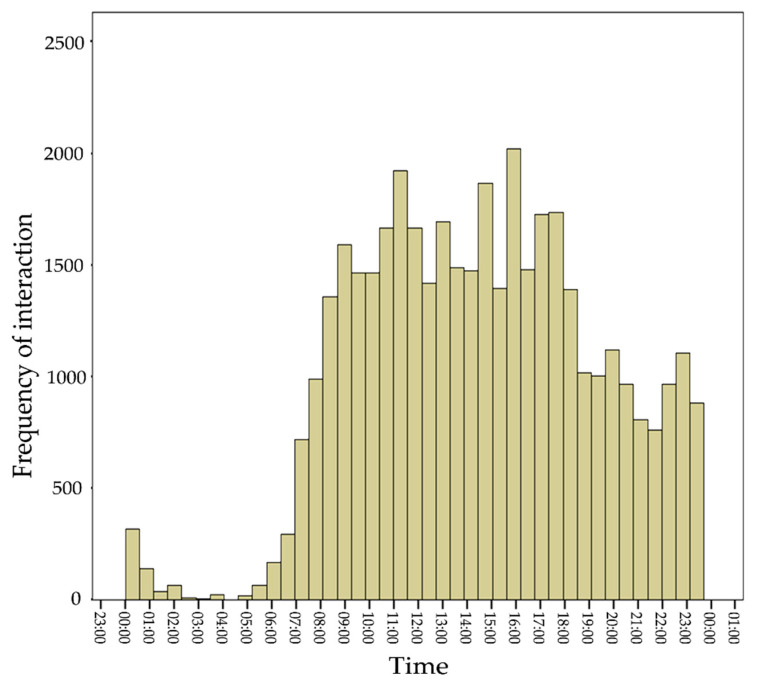
Frequency of interactions with eWALL over the day for all users.

There was a primary interest in the sleep application (21.9% of all interactions overall) and the application that showed a user’s physical activity (21.48% of all interactions).For the end-users with COPD and ARI, one other application stood out: The health application. For end-users with MCI, interest in this application was low, but replaced by a relatively high use of the cognitive games, which were specifically designed for this target population. In total, over 73% of all interactions with eWALL were centered on these four applications. The distribution of total interactions per application for ARI was much more evenly spread than for the other two sub-groups. Analyses of daily use of the platform showed that the activity, sleep, and health applications were used, on average, at least every other day by all users. The cognitive exercise and video exercise application were used at least every week. Finally, we examined how the interactions were distributed throughout the day (see Figure 5). The analysis shows that eWALL interactions occur evenly over the day, picking up between 07:00 and 08:00 in the morning and showing a stable number of interactions until 18:00 in the evening. The evening period between 19:00 and 00:00 shows slightly lower, but again stable use.

In order to increase the extractable information and integrate it with the results from the data log analyses, we conducted semi-structured interviews at the end of the intervention period. To optimize the user experience of the platform and to evaluate and resolve technical problems, an interview was organized to record the technical problems encountered by the participants; the following issues were mentioned more than once: activity tracker did not measure physical parameters correctly (steps, stairs, sleep, calories burnt); Bluetooth medical devices (for assessing blood saturation and blood pressure) did not synchronize with eWALL; eWALL was very slow or froze; the platform could not synch or lost its connection; domotics measurements were incorrect or did not work.

#### 3.3.1. Physical Rehabilitation Training

Most of the participants with ARI found the physical rehabilitation training too easy to do and quickly gave up, due to the lack of challenge. Some users decided not to use the physical training functionality of eWALL, as they already exercised in other ways. One participant suggested that the eWALL should provide the option of choosing exercises from a large repository, so that each user could assemble his/her own training program, geared towards individual goals. The participants with COPD would have preferred breathing exercises for pulmonary rehabilitation, alongside the exercises focusing on gaining strength, balance, and flexibility. One participant with ARI really liked the physical training videos and used them every day. In his opinion, the difficulty level was sufficient. And as he did not do any physical exercise before interacting with the eWALL, his activity level increased. All participants found the physical training application easy to use.

#### 3.3.2. Cognitive Rehabilitation Training

All MCI participants found the cognitive exercises enjoyable. All participants that tried out the cognitive rehabilitation training (including participants with COPD and ARI) found the game application fun and easy to use.

#### 3.3.3. Sleep Overview

Thirteen participants with COPD found it very interesting to receive information about their sleep. As a result, one participant with COPD tried to improve his sleep, but did not succeed. The other twelve stated that eWALL could not influence their behavior (4 times) or that eWALL lacked actionable advice on this matter (two times). One participant with ARI was very enthusiastic about the application and proper recognition of his sleep. Most participants with MCI (13 out of 15; six with and seven without sleeping problems) found the information about sleep interesting or very interesting, as well as clear.

#### 3.3.4. Health Monitoring

Nine participants with COPD measured their blood pressure and eight assessed their oxygen saturation. They did so as they were interested (7 times), they used this information while exercising (1 time), they wanted to check their health (2 times), or because someone told them to (1 time). Six of them indicated that the associated overviews on eWALL are nice. Two participants were rather negative. They found the information nothing new (1 time) or dull (1 time). The experience of the My Health book by the Danish and Austrian participants was influenced by the instability of the transfer of data. The health data was not available at all for some users and only partly available for the remaining users.

#### 3.3.5. Robot Notifications

Three participants with COPD negatively perceived the messages and alarms they received via the robot avatar. They found their content quite meaningless and irritating. Eight participants with COPD were positive, mentioning that it was a fun way to interact with eWALL (2 times) and that the messages were correct and lead to concrete actions on their part (4 times). Five out of the 15 participants with MCI mentioned that they received notifications via the robot and found these notifications interesting. The Danish participants did not express a lot of interest in interacting with the robot. Most of the users did not actively start an interaction with the robot. Two users found it too difficult to interrupt the interaction with the robot. One participant with ARI was able to comment on this feature, stating that the messages were motivating to be more physically active.

When we asked participants how they experienced the time with eWALL in their house, the majority was positive. The general experience with eWALL was found rewarding, stimulating, and interesting, by 27 participants. The most positive experiences with the platform were those linked with the monitoring and training of physical and cognitive capacity. The importance of cognitive training was confirmed by the interview results: eWALL was positively evaluated by 14 out of 15 MCI participants. The most negative experience was the instability of the platform (e.g., synchronization of devices, network problems) (29 out of 36).

### 3.4. Potential Clinical Effect

The score on the various domains of the SF-36 are presented in Table 6. For all outcomes there are no significant differences between pre-test and post-test scores (*p* > 0.087).

## 4. Discussion

In this study, we evaluated the acceptance, use, and potential impact on physical and mental health of eWALL, a platform for telemonitoring and telecontrol, based on pervasive health technologies. The developed framework provides healthcare services that enhance traditional medicine, exploiting the powerful applications of Internet and Technology. [1] eWALL was developed to support persons with age-related impairments (ARI), chronic obstructive pulmonary disease (COPD), and mild cognitive impairment (MCI) in managing their health and improving their physical and cognitive conditions, by means of collection and analysis of behavioral, environmental, and biometric data.

A key issue concerning pervasive mobile healthcare systems regards technology acceptance of different groups of users, given the different approach of single users with these devices and the variable perception about its utility. The need of further studies investigating this feature has been emphasized by Huzooree and colleagues, who reviewed and evaluated twenty recent studies monitoring patients affected by chronic diseases [5].

As a matter of fact, the acceptance of eWALL differed over the different study populations, i.e., ARI, COPD, and MCI, while the intention to use eWALL was influenced by the perceived ease of use and usability of the platform. The use of eWALL was intense in the first week of intervention and decreased, remaining stable, in the following weeks. The participation was undermined by technical issues, but several features were appreciated. In particular, all patient groups enjoyed receiving feedback on their sleeping behavior and physical activity. Persons with COPD and ARI also liked the option to have an overview of their health parameters (e.g., blood pressure). Persons with MCI on the other hand, valued positively eWALL’s cognitive training. The different attitude of the three study groups with respect to the Acceptance of eWALL was clearly demonstrated by the Likert scale. Subjects with ARI and MCI showed a more positive approach towards the technology if the system (all the scores are over the threshold of 4), while those affected by COPD proved less confident to use the device, and were overly critical and mistrustful of the technology. This could be due to several reasons. Firstly, persons with COPD found the interaction with eWALL and the platform functionalities poorly fitting with their wishes. For example, they missed COPD-specific exercises within the physical training. Secondly, despite a standardized installation protocol was used by all researchers in the four countries, the different attitude may have been generated by cultural distances (subjects with COPD were from The Netherlands and Denmark, while MCI participants were from Italy, and ARIs from Austria), or by the different approach used by the demonstrators during the installation phase. Furthermore, a possible interviewer bias in different groups may have determined groups discrepancies. However, different countries are likely to have different standards with respect to the use and quality of health technology, which influences people’s expectations and that should be considered when health technologies are proposed to study groups located in different geographic areas.

As regards the Intention to use the technology, backward linear regression analyses revealed three predictive factors: Trust in technology, Controllability, and Aesthetics. These variables drive the Intention to use the proposed technology, but a mediating role of the Perceived usefulness, and the Ease of use should be considered [35]. It worth to underline that low correlations and regressions values may be partially due to the heterogeneous responses to TAM questionnaires among patients groups; especially, despite the statistical significance inherent the model linking usefulness and ease of use to intention to use, linear regression returned coefficient determination R^2^ lower than expected. However, results highlight the importance of the interface design when advanced healthcare platforms are proposed: The intention to use a technology depends on the perceived usefulness and ease of use of such technologies, and these factors can be modulated, facilitating trust in technologies by stimulating persons’ awareness, and improving interfaces and graphic elements. Furthermore, even if the aesthetics domain could seem a not important factor, the appearance influences the enjoyment that the user may receive from the interaction with the platform, but not the possible intention to use eWALL. Indeed, as reported by Ziefle and colleagues, when facing healthcare technology, fun and aesthetics may not be necessary conditions to reach the expected results [36].This is the important lesson learned from this study, as it makes clear the need to take into account the complex network of relationships among features, strategic design, effective communication, and patients’ willingness.

Analysis of data logs clearly showed that, after the first week of intense use, most likely due to the novelty effect, the volume of interactions lowered but remained stable. This observation shows that participants were generally inclined to a constant use of the platform, appreciating the support in managing their condition or improving their health. Most participants gave positive feedback about sleeping behavior and physical activity, demonstrating that technologies serving as the home ‘health-hub’ should always provide behavioral descriptions. Other functionalities were more disease specific. Participants were not instructed to use a specific application, but they were invited to use the platform freely, following their needs and preferences. Indeed, it is quite interesting to notice the different approach to the platform experienced by target groups. The analysis of use showed how MCI participants focused more on cognitive activities, on activity applications and sleep behavior. The most used applications for the COPD target group are the Activity Application (23%), Sleep Application (22%) and Health Application (21%). Interestingly, participants with specific impairment focused freely on applications specifically designed for them. As mentioned above, participants with MCI used cognitive training far more than participants with ARI or COPD, which more often checked their physiological parameters (e.g., blood pressure, oxygen saturation). This result gives additional support to the proposal of conducting a fine-disease oriented evaluation of eHealth [37]. These technologies should be evaluated on their potential for the specific service they provide to different end-user populations.

It is not possible at this stage to draw conclusions about the impact of the platform on preserving functional capacity and improving quality of life, given the small sample size and the limited duration of the intervention. When considering the intervention period (from 2 to 6 weeks), it was clearly anticipated that the primary outcome of the study would be the acceptance of the system rather than stating improvements in cognitive or physical conditions. Indeed, it was quite unlikely that significant modulations of the cognitive, physical status or, perceived Quality of Life could be observed in such a short period. Obviously, we cannot rule out the possibility that eWALL does not have any positive effect on the health of end-users, having still in mind that facing progressive diseases such as those investigated in our experiment, the fundamental objective is to keep stable physical/cognitive conditions as long as possible, and the. However, the probability of a clinical effect of eWALL in persons with a chronic disease or age-related impairments is very high, given their use of the technology and comments during the interviews, and extending the study length is a priority goal.

Convincing potential users of these types of pervasive health technology to place such a big device in their homes for four weeks was a significant achievement, especially since they were informed that these ubiquitous technologies require a longer period of use before sorting any clinical effect. This condition presents a difficult situation for evaluators of pervasive health technologies, since they need to keep a balance among study length, participant burden, and the power of the studies to assess the possible effect of this technology on health parameters. Furthermore, a long lasting study could help to understand other long-term use effects, like annoyance and fatigue, that however patients could experience after several months of use of the platform. These are the greatest challenges for the planning and organization of future studies.

### Limitations

In our study, we included participants with different medical backgrounds, living in different countries. Since our sample was not big enough to simultaneously control for both factors, it is possible that differences among end-user groups were influenced by these parameters. Future studies focusing on the acceptance of pervasive health technology should take medical and cultural heterogeneity into account in their research models. The participants in our study regularly experienced technological problems while interacting with eWALL. This hindered their use of the platform or some of its features and may as well have affected their opinion and acceptance of the technology [1,16]. Pervasive health technologies in general are characterized by their low maturity level [5]. To create a solid and reliable pervasive health technology in a short time is challenging and requires the connection and integration of many technologies.

## 5. Conclusions

The present study shows that persons with COPD, MCI and ARI are in general open to interaction with pervasive technology, and that they are available to use it on a regular basis. In addition, they are interested to get more insights about their condition and they specifically interact with those features that are beneficial for them due to their medical background. Pervasive technology has the potential to make a difference in the life of people with chronic diseases or age-related impairments, although its technological immaturity may affect the results, especially as regards the management and clinical impact of the use.

In conclusion we suggest that a stable use of eWALL may stimulate participants with specific impairment to be more active with the applications designed, mainly for the purpose of the specific target group. This interaction can lead to beneficial effects including specific empowerment of users, promotion of cognitive and physical rehabilitation, self-management of health, sleep behavior monitoring and early detection of sleep disorders, physical activity reinforcement and cognitive stimulation.

A more extensive clinical trial with a more mature version of the technology and a longer time of interaction between the end-user and technology should therefore be conducted. Nonetheless, this study provides us with important advice for designing pervasive health technologies that are accepted by end-users and for setting up an evaluation of these innovations.

## Figures and Tables

**Table 1:** Main characteristics of the study participants.

	MCI (*n* = 15)	COPD (*n* = 23)	ARI (*n* = 10)
Gender	7 male/8 female	16 male/7 female	4 male/6 female
Age	71.9 (SD 0.9)	65.4 (SD 1.7)	66.4 (SD 1.8)
MMSE	23.7 (SD 0.1)	n.a.	n.a.
GOLD stage	n.a.	From 2 to 4	n.a.
Days of eWALL use	29.6 (SD 2.7)	22.8 (SD 2.1)	20.1 (SD 2.2)

MCI: Mild Cognitive Impairment; COPD: Chronic Obstructive Pulmonary Disease; ARI: Age Related Impairment; MMSE: Mini-Mental State Examination.

**Table 2:** Reliability scores of rating scales for Technology Acceptance factors.

Scale	No of Items	Cronbach’s Alpha
Enjoyment	4	0.86
Aesthetics	10	0.96
Control	3	0.76
Trust in technology	4	0.98
Perceived usefulness	6	0.83
Ease of use	4	0.93
Intention to use	3	0.93

**Table 3:** Mean scores and standard deviations for Technology Acceptance factors.

Scale	ARI	COPD	MCI	Overall
Enjoyment	4.80 (1.08)	3.14 (1.24)	5.12 (1.09)	4.19 (1.48)
Aesthetics	5.04 (0.54)	2.98 (1.28)	5.57 (0.86)	4.34 (1.61)
Control	5.67 (0.53)	2.54 (0.67)	4.64 (1.00)	3.85 (1.46)
Trust in technology	4.40 (0.72)	2.61 (1.23)	4.98 (1.78)	3.85 (1.81)
Perceived usefulness	4.04 (1.05)	3.45 (1.24)	4.99 (0.76)	4.17 (1.24)
Ease of use	6.00 (1.16)	2.34 (1.06)	5.58 (1.13)	4.20 (2.00)
Intention to use	4.40 (1.91)	2.75 (1.76)	5.18 (1.19)	3.99 (1.91)

**Table 4:** Pearson’s correlation coefficient among Technology Acceptance factors.

	Enjoyment	Aesthetics	Control	Trust	Perceived Usefulness	Ease of Use	Intention to Use
Enjoyment	1						
Aesthetics	0.85 *	1					
Control	0.63 *	0.72 *	1				
Trust	0.62 *	0.76 *	0.63 *	1			
Perceived usefulness	0.55 *	0.60 *	0.53 *	0.68 *	1		
Ease of use	0.65 *	0.80 *	0.86 *	0.64 *	0.55 *	1	
Intention to use	0.67 *	0.68 *	0.53 *	0.69 *	0.73 *	0.59 *	1

* *p* < 0.001.

**Table 5:** The distribution of the use of eWALL applications for all participants and each sub-group (top three in bold).

	All	COPD	MCI	ARI
Sleep book	**21.90%**	**22.13%**	**21.65%**	**21.73%**
Activity book	**21.48%**	**22.81%**	**22.98%**	**16.47%**
Health book	**15.10%**	**21.01%**	3.46%	**18.18%**
Cognitive games	14.51%	7.47%	**28.66%**	10.49%
Main screen	8.33%	10.37%	7.89%	4.45%
Calendar	5.56%	5.06%	4.21%	8.56%
Domotics	5.28%	3.86%	3.20%	11.28%
Physical training	4.74%	4.69%	3.45%	6.63%
Reward app	1.07%	0.71%	1.82%	0.80%
Help	1.04%	1.13%	1.28%	0.51%
Fall prevention	0.99%	0.77%	1.41%	0.91%

**Table 6:** Overview of SF-36 domain scores for all participants and the three groups before (pre-test) and after use (post-test) of eWALL in mean (and standard deviation).

	All Participants (*n* = 36)		ARI (*n* = 5)		COPD (*n* = 16)		MCI (*n* = 15)	
	Pre-Test	Post-Test	Pre-Test	Post-Test	Pre-Test	Post-Test	Pre-Test	Post-Test
Physical functioning	59.3 (26.6)	58.5 (24.4)	76.0 (17.5)	70.0 (25.0)	45.3 (28.0)	45.0 (24.1)	69.7 (19.5)	70.0 (16.8)
Role limitations due to physical health	49.3 (41.5)	50.0 (37.7)	85.0 (33.5)	55.0 (51.2)	30.9 (40.0)	41.2 (38.5)	58.3 (36.2)	58.3 (32.3)
Role limitations due to emotional problems	61.1 (37.3)	66.5 (36.9)	80.0 (44.7)	86.7 (30.0)	68.6 (40.0)	80.4 (39.2)	46.3 (27.5)	44.0 (23.9)
Energy/Fatigue	56.1 (15.6)	56.4 (11.8)	63.0 (13.5)	61.0 (14.3)	58.2 (18.9)	59.7 (13.4)	51.3 (10.9)	51.0 (6.0)
Emotional well being	74.6 (13.8)	74.2 (14.3)	68.0 (10.2)	69.7 (6.7)	83.3 (12.1)	84.2 (13.0)	66.9 (11.2)	64.3 (9.2)
Social functioning	72.5 (18.9)	72. 8 (19.5)	92.5 (16.8)	90.0 (16.3)	71.1 (19.2)	73.4 (20.9)	67.3 (15. 5)	66.5 (16.1)
Pain	77.5 (22.4)	73. 8 (23.8)	77.1 (8.5)	67.4 (21.7)	81.2 (29.2)	75.2 (30.4)	73.4 (16.0)	74.5 (15.9)
General Health	46.7 (17.7)	44.9 (21.2)	67.0 (13.5)	74.0 (14.7)	40.6 (16.8)	35.3 (19.4)	46.7 (15.4)	46.1 (15.7)
